# Topical carnosine gel improves intermittent high-intensity exercise performance in world-class rugby sevens players

**DOI:** 10.1080/15502783.2025.2550311

**Published:** 2025-08-27

**Authors:** C. Martyn Beaven, Carl James, Daniel T. McMaster, Nic Brockelbank

**Affiliations:** a University of Waikato, Te Huataki Waiora School of Health, Tauranga, New Zealand; b Department of Sports and Health Sciences, Academy of Wellness and Human Development, Faculty of Arts and Social Sciences, Hong Kong Baptist University, Hong Kong, SAR China; c All Blacks Sevens, New Zealand Rugby, Wellington, New Zealand

**Keywords:** Transdermal, buffering, peak power, athletic performance

## Abstract

**Background:**

The topical application of a carnosine gel may increase intramuscular carnosine concentrations and thereby improve exercise performance. This study investigated the effect of a topical carnosine gel on intermittent high-intensity exercise performance in rugby sevens players.

**Methods:**

Seven world-class rugby sevens players (body mass: 97.5 ± 4.1 kg; 1,452 International caps) completed two performance tests in a counterbalanced, crossover, triple-blind design. Players applied either 10 mL of a topical carnosine gel (CAR) or an ultrasound placebo gel (PLA) 40 minutes before undertaking 12 intermittent sprints on a cycle ergometer, interrupted by a 2-minute break after Sprint 6. The activity profile was 24 s cycling at 3 W/kg, 6 s at maximal intensity sprint, followed by a 30s rest. Average power, peak power, and HR were recorded for every 6 s sprint. RPE was recorded at the end of Sprint 6 and Sprint 12.

**Results:**

For peak power, two-way ANOVA revealed time (*p* = 5.49 × 10^−9^) and treatment effects (*p* = 1.44 × 10^−7^). Following CAR, peak power output was higher in Sprint 2 (1433 vs 1332 W; *p* = 0.048; *d* = 0.99; *large*), Sprint 4 (1347 vs 1244 W; *p* = 0.043; *d* = 0.74; *moderate*) and Sprint 7 (1426 vs 1270 W; *p* = 0.025; *d* = 0.98; *large*) compared to PLA. For mean power output, HR, and RPE there were time effects but no treatment effect (*p* = 0.211 to 0.847).

**Conclusions:**

Topical carnosine gel improved power production in world-class rugby sevens players. Future research should determine whether carno- sine gels increase intramuscular carnosine concentrations to support the observed improvements in anaerobic performance during high-intensity exercise.

## HIGHLIGHTS

- We demonstrate initial findings regarding the efficacy of a transdermal carnosine gel to impact repeated, high-intensity exercise in world-class rugby sevens players (+5–10%).
- The acute positive performance effects occurred rapidly without the need for a loading phase typical with β-alanine supplementation.
- The positive performance effects were evident during the initial sprint and thus, appear to be independent of intracellular buffering capability.
- The transdermal application would be of benefit to individuals whose diet predisposed them to low carnosine levels, such as vegetarians.

## 1. Introduction

During high-intensity exercise, the accumulation of H^+^ ions decreases intracellular pH. This acidification may impair performance by limiting the resynthesis of high-energy phosphates and inhibiting the rate of anaerobic glycolysis [1]. To mitigate these effects, intracellular and extracellular buffer systems act to reduce the accumulation of H^+^ ions and delay the onset of fatigue [2]. Carnosine (β-alanyl-L-histidine) is a naturally occurring histidine-containing dipeptide molecule and is a strong intracellular buffer found in high concentrations within the cytosol of skeletal muscle [3]. Thus, increasing carnosine concentrations can enhance pH buffering and improve high-intensity exercise performance [4,5]. An increase in carnosine concentrations has also been shown to counteract reduced Ca^2+^ sensitivity, which could alleviate the decline in performance during fatiguing exercise [6]. Additionally, an increase in carnosine concentrations may improve myosin-ATPase activity and increase the maximal amount of adenosine triphosphate re-synthesized via anaerobic metabolism [7].

Many nutritional supplements contain high levels of carnosine to increase muscle carnosine concentrations, thereby supporting high-intensity exercise performance. However, due to the highly active serum carnosinase enzyme, the ingestion of carnosine is susceptible to hydrolysis and even relatively high doses of carnosine (60 mg.kg^−1^ bodyweight) are often rapidly degraded leading to individual variability in plasma carnosine levels [8]. Therefore, alternative methods of enhancing muscle carnosine concentrations have been investigated, such as β-alanine supplementation. β-alanine is a naturally occurring amino acid which is a requisite for carnosine synthesis [9]. Four weeks of oral β-alanine supplementation at a rate of 4–6 g.day^−1^ may raise intramuscular carnosine levels by ~ 60% [10] and a meta-analysis concluded that supplementation had an ergogenic effect on exercise lasting between 1–6 minutes [11]. Moreover, shorter duration events and events with repeated high-intensity efforts that elevate blood lactate may be more relevant given the pH buffering capacity of carnosine. However, since β-alanine supplementation has a loading period of at least 2–4 weeks [12], a more efficient method to increase muscle carnosine concentrations is desirable.

The topical application of carnosine directly to the skin may allow carnosine to be absorbed into the bloodstream via a transdermal drug delivery system [13]. In an equine model, transdermal application of carnosine increased muscle carnosine concentrations by up to 76%, offering a different methodology to support high-intensity exercise performance [14]. Sharpe and Macias [15] found that the topical application of a carnosine gel significantly improved the average time to complete three all-out 1000 m time trials by 4% (248.8 ± 9.6 vs 239.7 ± 12.6) in international-level soccer players, compared to a placebo gel. However, a more recent study, found no statistically significant improvements in glycolytic capacity or Wingate sprint performance following the topical application of carnosine in amateur cyclists [16]. Notwithstanding, there is some evidence that the topical application of a carnosine gel may serve as a performance ergogen. Gel application provides tangible practical benefits over existing supplementation methods which require prolonged loading periods.

Strategies to increase carnosine concentrations and enhance muscle buffering capacity have relevance for rugby sevens players. Due to the short duration of games (2 ×7 min halves) and higher relative pitch area per player than any other running-based team sport, high locomotor activity and frequent high-intensity repeated performances are observed during sevens matches [17]. Players spend ~ 14% of matches undertaking high-intensity running (18–20 km⋅h^−1^) or sprinting ( > 20.1 km⋅h^−1^) [18]. The anaerobic requirements of rugby sevens are highlighted by recent research showing ~ 43% of blood lactate samples exceeded 10 mmol⋅L^−1^, and two samples exceeded 19 mmol⋅L^−1^ following international matches [19]. These results suggest that the glycolytic energy system plays an important role in rugby sevens performance and that considerable metabolic stress occurs during a rugby sevens match [20]. Thus, it seems feasible that rugby sevens players will benefit from increased muscle carnosine concentrations and associated intramuscular buffering capacity following carnosine supplementation.

However, there is currently no evidence on the topical application of a carnosine gel and its efficacy as an ergogenic aid for rugby sevens players. Given earlier contrasting findings, the aim of this pilot study was to investigate the application of a topical carnosine gel and its effects on intermittent high-intensity exercise performance in world-class rugby sevens players. It was hypothesized that the application of a topical carnosine gel would increase both peak power output and repeated sprint performance, during repeated bouts of high-intensity exercise.

## 2. Materials & methods

### 2.1. Participants

Twelve male rugby sevens players provided written informed consent to participate in this study. The players were defined as world-class (Tier 5) based on an established participant classification framework [21]. The study protocol was approved by an institutional Ethics Committee and followed the ethical principles laid out by the Code of Ethics of the World Medical Association (Declaration of Helsinki). Before participating in the study, participants were familiarized with the study and experimental protocol. Of 12 participants, five withdrew due to injury unrelated to the experimental protocol, other commitments, or competition demands. Seven participants completed the study (30.25 ± 3.65 y, 190 ± 4.41 cm, and 97.45 ± 4.09 kg), with a combined 1,452 International caps.

### 2.2. Research design

This was a triple-blind, placebo-controlled, and counterbalanced crossover study. To ensure the treatment and placebo were blinded, an independent researcher who was not involved in the data collection separated the two products into unlabeled yellow and red bottles. Players were informed that two new products were being trialed; however, one bottle contained the carnosine gel (CAR: LactiGo™, Outplay Inc, Las Vegas, NV) and the other contained the placebo gel, which was a clear ultrasound gel (PLA: Aquasonic 100, Parker Laboratories Inc, Fairfield, NJ). All statistical analysis was performed prior to unblinding. During the first testing day, and in a counterbalanced design, participants were randomly allocated to receive either the CAR or PLA gel by block randomization to ensure a balance in sample size across treatments. Seven days later, the treatments were switched, and the experimental protocol repeated. Four participants received CAR on testing day one, and three received CAR on testing day two. All performance tests were completed between 1:00 PM and 4:00 PM after the team’s gym session. After the completion of all testing, the nature of the placebo deception was disclosed to the participants.

### 2.3. Intervention

Forty-five minutes before the performance test participants manually applied 10 mL of either the CAR or PLA gel to each leg (thigh and calves). The quantity and timing of the gel application were based on the manufacturer’s recommendation for the LactiGo™ gel. The LactiGo™ carnosine gel comprises water, glycerin, magnesium sulfate, 1.25% menthol, and a proprietary carnosine complex. The PLA gel had 1.25% menthol added to mimic the odor and stated concentration of the active ingredient in the CAR gel.

### 2.4. Performance test protocol

The performance test was based on the protocol previously used by Fenemor and colleagues [22], designed to simulate the work-to-rest ratio of a rugby sevens match and to assess performance change in world-class rugby sevens players. A coefficient of variation of 4.2% and a smallest worthwhile change of 41.8 W have previously been reported for peak power during similar exercise in male rugby players performing repeated sprints [23]. During the first trial, before the start of the test, the participant’s stature (cm) and body mass (kg) were recorded. Body mass was used to calculate target zones for 2, 2.5, and 3 W.kg^−1^ for exercise intensities during the performance test. All testing was completed on the same, calibrated cycle ergometer (Wattbike Ltd, Nottingham, UK), with the seat, and handlebar positions standardized for each player across testing sessions. The protocol consisted of a warm-up, followed by 12 intermittent sprints, separated by a 2-min half-time break after Sprint 6. The warm-up took the following structure: 2-min cycling at 2 W.kg^−1^, 2-min cycling at 2.5 W.kg^−1^ and 30 s cycling at 3 W.kg^−1^. This was followed by a 2-min rest before the start of the intermittent sprint protocol. The intermittent sprint section consisted of 12 repeated efforts of 24 s cycling at 3 W/kg, 6 s maximal intensity, and a 30s rest.

During the test, heart rate (HR) was measured using a Polar H10 chest strap monitor (Polar Electro Oy, Kempele, Finland). Ratings of perceived exertion (RPE) were recorded using the Borg Rating of Perceived Exertion Scale [24]. The RPE, HR, and average power were recorded after each stage of the warm-up. The RPE was recorded at the end of Sprint 6 and Sprint 12. Average and peak power and HR were recorded after each 6s sprint.

### 2.5. Statistical analysis

Data were grouped as YELLOW or RED depending on the color of the unlabeled bottles used for dispensing the gels and results were analyzed for HR, RPE, average 6 s sprint power (MP), and peak 6s sprint power (PP). Half-time recovery for average and peak power was calculated as the change in power between Sprints 7 and 6. Data were analyzed using a two-way ANOVA (Time: Sprint number ×Treatment: YELLOW and RED). When a treatment effect was identified, a *post-hoc* t-test was used to determine the difference between the two treatments (YELLOW and RED). The difference (YELLOW – RED), ± standard deviation, percentage change, and effect size were calculated for every data point. Statistical analyses were performed on log-transformed data to account for non-uniformity of errors [25]. The statistical significance threshold level was set at an alpha level of 0.05. To interpret the effect size between the two treatments (YELLOW and RED) the magnitude of the effect was determined and expressed as mean differences ± standard deviation and Cohen’s *d* was calculated with a 90% confidence level. The thresholds used to describe effect sizes were *trivial* ( < 0.20), *small* (0.20–0.49), *moderate* (0.50–0.79), and *large* (≥0.80) [26]. An effect was deemed *clear* if the confidence interval did not overlap the thresholds for both small positive and small negative effects. Non-significant but *clear* differences are herein denoted as substantial [27].

## 3. Results

All data were normally distributed after the requisite transformation. No side-effects such as paresthesia were reported in either testing session. Successful blinding was indicated with four of the seven players reporting feeling that their performance was improved in the placebo treatment (χ^2^ = 0.143; *p* = 0.7055).

### 3.1. Peak sprint power

**Figure 1.**
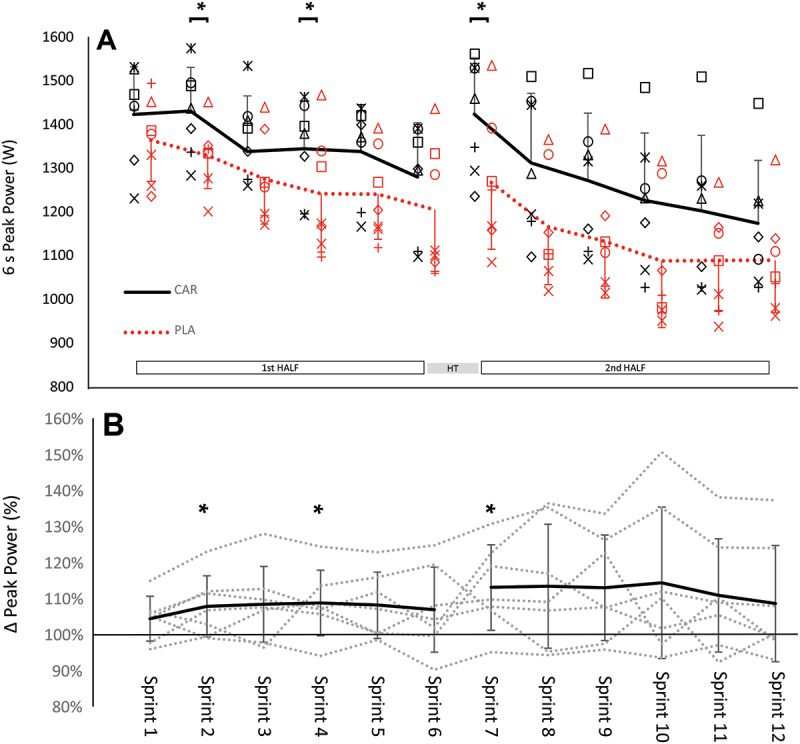
A. Peak 6s Sprint power output following the topical application of the carnosine gel (CAR) and the ultrasound placebo gel (PLA). Individual data symbols represent individual data from each player. HT: 2 min half -time rest; *: *p* < 0.05. 1B. Percentage difference between the CAR and PLA conditions. Dashed lines represent individual players with the solid line representing the mean difference. *: *p* < 0.05. All error bars represent standard deviations.

The two-way ANOVA revealed statistically significant time (*p* = 5.49 ×10^−9^) and treatment effects (*p* = 1.44 ×10^−7^). Overall, for the time effect, peak power decreased from Sprint 1 to Sprint 12 by 261.9 ± 123.2 W (*p =* 3.89 × 10^−6^). Following unblinding, post-hoc analyses showed that for Sprints 2, 4 and 7 the difference in peak power between the two treatments was statistically significant (Figure 1 (A,B) *p* < 0.05). Specifically, the peak power during CAR treatment was greater during Sprint 2 (1433.3 vs 1332.0 W; Δ + 101.3 ± 81.0 W; *p* = 0.048; *d =*0.99; *large*), Sprint 4 (1347.0 vs 1244.4; Δ + 102.6 ± 82.1 W; *p* = 0.043; *d* = 0.74; *moderate*), and Sprint 7 (1426.3 vs 1270.3; Δ + 156.0 ± 106.2 W; *p* = 0.025; *d =*0.98; *large*) when compared to PLA. The differences in peak power between the CAR and PLA treatment were *large* during Sprints 3, 8, 9, and 10 (+101.1 to 145.6 W; *d* = 0.81 to 0.91), *moderate* during Sprints 5 and 11 (+97.3 and + 113.3 W *d* = 0.79 and 0.67), and *small* during Sprints 1 and 6 (+58.9 and + 74.4 W; *d* = 0.49 and 0.49). These values all exceeded the smallest worthwhile change 41.8 W reported during repeated 6-second sprints identified *a priori* in a male rugby population [23]. Sprint 12 displayed neither a statistically significant nor substantial difference in peak power between the two treatments. The half-time recovery of peak power was + 144.1 ± 97.6 W for the CAR treatment and + 62.6 ± 81.9 W for the PLA treatment (*p =*0.193; *d =*0.71; *moderate*).

### 3.2. Mean sprint power

The two-way ANOVA revealed a statistically significant effect of time (*p* = 5.98 ×10^−19^), but not treatment (*p* = 0.211). Overall, for the time effect, mean power decreased from Sprint 1 to Sprint 12 by 284.9 ± 113.9 W (*p =*3.86 ×10^−7^). Despite, no significant differences between the two treatments, mean power was substantially higher in the CAR compared to the PLA treatment in Sprint 10 (992.0 vs 940.7 W; Δ + 51.3 ± 48.2 W; *p* = 0.079; d = 0.57; moderate) and Sprint 11 (982.7 vs 938.0 W; Δ + 44.7 ± 37.0 W; *p* = 0.069; d = 0.39; small). For the remaining sprints, as well as half-time recovery, there were no *clear* nor statistically significant differences between the two treatments for mean power.

### 3.3. Heart rate

The two-way ANOVA revealed a statistically significant time effect (*p* = 3.12 ×10^−15^) but no treatment effect (*p =* 0.228). Overall, for the time effect, HR increased from Sprint 1 to Sprint 12 by 20.8 ± 9.5 bpm (*p =*4.26 ×10^−7^) with an average of 164.5 ± 7.5 bpm after Sprint 12.

### 3.4. Ratings of perceived exertion

The two-way ANOVA revealed a statistically significant time effect (*p* = 9.88 ×10^−17^) but no treatment effect (*p =*0.847). Overall, for the time effect, RPE increased from post-warm-up to Sprint 12 by 7.1 ± 1.9 units (*p =*1.41 ×10^−10^) with an average of 18.7 ± 0.9 units after Sprint 12.

## 4. Discussion

This study investigated the topical application of a carnosine gel on intermittent high-intensity exercise performance in world-class rugby sevens players. The findings herein indicate that the topical application of a carnosine gel can significantly improve peak power output during high-intensity intermittent exercise performance in world-class athletes. If carnosine gel has increased muscle carnosine levels to a similar magnitude as in equine models, these small but meaningful positive performance effects during the first sprint effort indicate an alternative physiological mechanism to previous carnosine applications, which enhance performance during later, repeated high-intensity efforts.

In this pilot study, peak power output increased by 4.1% in Sprint 1, with the largest increase being 11.4% in Sprint 10. The increase observed in Sprint 10 is larger than the previously seen 3% increase in peak power output during three consecutive Wingate tests in trained males following the supplementation of combined carnosine and anserine [28] and the 6% increase in lower-body peak power in national-level boxers following β-alanine supplementation [29]. Future work should determine whether increased muscle carnosine concentrations are responsible for the acute increases in peak power output observed in our elite athletic cohort, as previous research has indicated a link between carnosine concentrations and peak power output [30]. Topical carnosine gel application transports carnosine across the skin via passive transdermal transport through the stratum corneum using a positively charged carnosine-magnesium complex, thereby allowing carnosine to be absorbed into the bloodstream and increasing muscular carnosine concentrations [14,31,32]. Thus, the application of a topical carnosine gel may lead to increases in peak power output of a similar magnitude to preexisting nutritional supplements and loading regimens.

The results of this current study suggest that a topical carnosine gel can improve repeated high-intensity exercise performance in world-class team sports players. This contrasts with a previous study by  Harnish and Miller [16] whereby a topical carnosine gel did not improve repeated high-intensity exercise performance in amateur cyclists. This discrepancy may be related to training history. Sharpe and Macias [15] demonstrated that topical carnosine improved anaerobic capacity during a ~4 minute running test in international soccer players. Compared to endurance-trained cyclists and triathletes, rugby sevens and soccer players typically undertake repeated, high-intensity exercise. Therefore, their muscle phenotype may be impacted differently using a topical carnosine gel. Previous research has suggested that athletes who excel in short, repeated, high-intensity exercise tend to have lower serum carnosinase activity and therefore may be more responsive to the potential ergogenic benefits associated with increased carnosine concentrations [33]. It is also noteworthy that the 1-s peak power wattages reported by Harnish and Miller [16] fall outside the range of acceptable accuracy (250 to 700 W) reported by Zadow and colleagues for the Wahoo Kickr device [34], and the greater variability in recorded power at wattages > 750 may have contributed to the lack of significant findings. Therefore, we advocate future research considers specificity of exercise and training history when investigating carnosine supplementation.

In the current protocol, there were no significant differences in mean power output between the treatments. This observation is consistent with observations from cyclists undertaking repeated Wingate protocols [16]. However, there was a significant increase in peak power output throughout the test. These acute effects demonstrate an increase in anaerobic work capacity following the topical application of the carnosine gel that we contend may not be solely attributed to the traditionally accepted intramuscular muscle buffering capability of carnosine. Greater carnosine concentrations may enhance anaerobic work capacity by improving myosin-ATPase activity and increasing the maximal amount of adenosine triphosphate re-synthesized via anaerobic metabolism during a specific mode of short, high-intensity exercise [7]. Increased intramuscular carnosine concentrations may also support Ca^2+^ handling and improve diffusive coupling [35], which enhances skeletal muscle force production and may improve high-intensity exercise performance. Herein, the topical application of a carnosine gel produced a demonstrable improvement in anaerobic work capacity during repeated bouts of high-intensity exercise. Furthermore, these improvements were of similar magnitude to those reported in a similar cohort when assessing the effectiveness of a heat acclimation protocol [22]. These improvements are likely beneficial for performance across a range of sports that rely on anaerobic energy pathways, including rugby sevens, football, ice hockey, field hockey, and basketball.

The results from this pilot study also demonstrate that a topical carnosine gel acts rapidly (i.e. from 45 min prior), thus eliminating the need for the loading period lasting weeks that is typically required to increase carnosine concentrations [10]. A topical carnosine gel would likely be absorbed into the interstitial fluid before being taken up by the muscle [36], bypassing the potential for breakdown via the highly active serum carnosinase enzyme [8]. Thus, topical application may have the potential to provide an efficient method to increase intramuscular carnosine concentrations compared to nutritional supplements; however, no definitive statement can be made as no direct comparisons were made between oral dosing and topical application. Nevertheless, the transdermal delivery method we assessed is time efficient and may be practically implemented across a range of sports, as well as in individuals whose diet predisposes them to lower carnosine levels, such as vegetarians [37].

The main limitation of this study is that no measures of serum, interstitial fluid, or muscle carnosine levels were analyzed. However, equine research does support the feasibility of raising muscle carnosine content via transdermal delivery [14]. Circulatory lactate values were similarly not assessed, which precludes insight into the mechanistic underpinnings of the performance changes observed. We acknowledge that this pilot study was conducted on a relatively small male-only cohort. With respect to the sample size, we assessed the entire population of Tier 5 players meeting inclusion criteria at the national training center. Such an elite cohort typically exhibits greater consistency in performance [38], and are more insensitive to performance enhancements due to their training status and a potential ceiling effect. As such, observed small worthwhile changes may be highly meaningful. It remains to be established how the topical application of a carnosine gel may improve high-intensity exercise performance in women or non-elite players. Previous research has suggested that women tend to have lower levels of carnosine so may see a larger increase in intramuscular carnosine concentrations [39]. A further limitation is that players’ diets and baseline muscle carnosine levels were not monitored before the performance test and it can not be concluded whether circulating levels of carnosine or other dietary interventions impacted the observed results. For example, no record was made as to whether specific athletes were vegetarian or adhering to diets high in red meat that would have low and high levels of carnosine intake, respectively.

Nonetheless, the results from this current triple-blind, counter-balanced, crossover study involving highly trained world-class rugby players, provide compelling evidence for the efficacy of a topical carnosine gel to improve repeated, high-intensity exercise. The practical implications of this study relate to the novel topical application of a carnosine gel and its efficiency as an ergogenic aid for a range of other sports that have a high anaerobic component as well as the need for repeated bouts of intermittent high-intensity exercise, such as 15-a-side rugby, football, ice hockey, and basketball.

## 5. Conclusion

A topical carnosine gel significantly improved intermittent, high-intensity exercise performance in world-class rugby sevens players. Given the timing of the peak power enhancement, (i.e. from the first sprint) it appears unlikely that the entirety of the observed increases in exercise performance can be explained by the muscle buffering capacity of carnosine, which would take longer to present. The topical application of a carnosine gel may provide a time-efficient method to enhance peak power performance. Future research should investigate any effects on intramuscular carnosine concentrations. More research investigating the mechanisms underpinning the observed performance enhancement from topical carnosine application is required to understand how different timing and dosing protocols may affect anaerobic performance, lactate concentrations, and intramuscular carnosine concentrations.
